# Long Suffering

**Published:** 1889-11-02

**Authors:** 


### Words of Consolation.

### LONG-SUFFERING.

*(For Reading to the Sick.)*

Long-suffering is the gift of the Holy Spirit, which enables us to receive all chastisement from God's hands without being paralysed or sinking under the sorrows and afflictions which He sends upon us. Job complains that man is born to sorrow as the sparks fly upwards, and truly misery is his inheritance since Adam lost his birthright of happiness by the fall. It is only God's mercy and compassion which can put us back into the Paradise of God. He has given His only Son to save us, but we must be trained by suffering before we shall be fit to dwell with the Most High. Long-suffering is the virtue which helps us in our warfare against the enemy of souls; it is that part of charity or love which beareth all things and endureth all things; it gives the Christian power to bear his chastisements, and to use them for his benefit instead of being irritated, fretted, and put out by them.

We should early realise that the strength which is necessary to make us God's soldiers and servants to our lives' end (direct supplies of which we receive in the appointed means of grace, baptism, confirmation, and holy communion) is increased by using our trials and sufferings to purify, chasten, and brace our souls. What benefit is it to sit down and lament that everything is against us, that our surroundings are hateful, that our lot in life is just what we should like to avoid, that our crosses would be lighter if we might choose them for ourselves? O, foolish hearts that thus think! Has not the potter power over the clay to make one vessel to honour and another to dishonour, and must not the Divine Artificer be the best Judge of the means by which He will mould our souls into the best form they are capable of attaining to? We are exposed to temptations and trials in order that we may be strong in faith and perfect. One side of our character is weak, we are irritable, or fiery and passionate. To counteract this, God places us among those who chafe us at every turn, in order that by controlling our tempers we may become gentle and meek. Moses is a beautiful example of this. In his early years he was hasty and impetuous; he killed the Egyptian who was oppressing his countryman, and in his dealings with the chosen people of God, the Israelites, his zeal outran his discretion, and, as we read in the Psalms, "he spake unadvisedly with his lips." For this he was punished by the Almighty, but in the end long-suffering triumphed. His nature was purified and strengthened, though it took nearly forty years of trial before it could be said of him, that the man Moses was very meek above all the men upon the earth. Bishop Milman says, "You can hurry man, but you cannot hurry God." He is patient because He is eternal. When we have to endure slights or affronts put upon us or feel ourselves passed by or overlooked let us remember our Lord Jesus Christ, who stood alone, often misunderstood by His friends and earthly relations; His disciples whom He had formed and even those whom He had benefited seeming the soonest to forget and desert Him. Above all, we must keep close to God. All patient endurance is a gift of the Holy Spirit. "Our nature is to be impatient, nasty, fretful, weary, rebellious, but long-suffering is above Nature—it is a fruit of the Spirit."

